# Applying the Human-Centered Innovation Biodesign Framework to the Development and Piloting of a Program to Mitigate Risk for Cognitive Decline Among Historically Underrepresented Individuals: Case Study

**DOI:** 10.2196/64930

**Published:** 2025-10-15

**Authors:** Rebecca Lassell, Ada Metaxas, Katherine Wang, Sara Hantgan, Prabhat Gottipati, Sarah Zwerling, Triana Pena, Chava Pollak, Laura Gitlin, Sunit Jariwala

**Affiliations:** 1 Department of Health and Wellness Design School of Public Health Indiana University Bloomington, IN United States; 2 Regenstrief Institute Indiana University Center for Aging Research Indianapolis, IN United States; 3 Department of Chemistry Princeton University Princeton, NJ United States; 4 Albert Einstein College of Medicine Bronx, NY United States; 5 Michigan Medicine Department of Computational Medicine and Bioinformatics University of Michigan School of Literature, Science, and the Arts University of Michigan School of Information Ann Arbor, MI United States; 6 Lewis Katz School of Medicine Temple University Philadelphia, PA United States; 7 College of Human Ecology and majoring in Human Development Cornell University Ithaca, NY United States; 8 Department of Neurology Stony Brook Medicine Long Island, NY United States; 9 College of Nursing and Health Professions Drexel University Philadelphia, PA United States; 10 Department of Medicine Albert Einstein College of Medicine Bronx, NY United States; 11 Division of Allergy/Immunology Albert Einstein College of Medicine Bronx, NY United States

**Keywords:** user-centered design, medical innovation, Alzheimer’s disease, dementia, lifestyle intervention, nature-based, innovation, human-centered, biodesign, cognitive, cognition, cognitive decline, Alzheimer’s, gaps, human-centered design, physical activity

## Abstract

**Background:**

Physical inactivity is a modifiable risk factor for dementia. Past physical activity interventions often overlook the voices of the end user in the design process, particularly minoritized groups living with dementia or memory challenges. To develop physical activity interventions, we use the principles of human-centered design.

**Objective:**

We applied human-centered design using the innovation biodesign framework to develop a physical activity intervention, Nurturing Aging Through Uplifting Activities in a Restorative Environment (NATURE) program for minoritized individuals as a use case.

**Methods:**

The innovation biodesign framework has three domains: (1) problem space, (2) invention, and (3) solution space. Each domain includes several activities. The problem space involves a needs assessment, needs screening, evidence-based literature review, review of existing models of programs, and iterative feedback from partners, leading to an invention. The solution space encompasses the implementation and validation of the invention and outcomes. We applied this framework in 3 steps: (1) identifying the problem: we used data points from multiple sources to identify needs and mapped them onto the problem space. These sources included reviews of the literature to identify existing interventions, findings from other nature programs to surmise gaps, and focus groups to iteratively identify unmet needs. (2) Designing the invention: we developed NATURE with Hispanic or Latino people with memory challenges and identified their preferred outcomes. (3) Mapping the pilot study. We added the study protocol and planned outcomes to the solution space.

**Results:**

In step 1, three evidence-based programs guided the development of NATURE to address physical inactivity and related risks of decreased well-being and dementia. We received 50 referrals for focus group participants, 22 were eligible and completed consent, and 21 (n=6 Hispanic or Latino people with memory challenges and care partners, n=8 outdoor professionals, and n=7 health care providers) participants completed the focus groups. We received feedback from participants on local nature activities, program frequency, duration, and delivery mode, a referral pathway, and outcomes using 5 focus groups and 2 interviews. In step 2, the 12-week NATURE program was developed to promote an active lifestyle and well-being, using nature activities that a person enjoys. NATURE accounts for a person’s preferences, needs, and daily situation and includes 4-6 sessions with 2 phone check-ins. Preferred outcomes were well-being, sleep, and social connections. In step 3, we mapped the plan to pilot NATURE using activity tracker technology to measure sleep, heart rate, and activity (well-being), and validated questionnaires.

**Conclusions:**

The framework provided a systematic approach for mapping the development of NATURE to address the needs of Hispanic or Latino people with memory challenges, using human-centered design principles. Application of the framework can be a helpful tool to map the development of other interventions for minoritized populations.

**Trial Registration:**

ClinicalTrials.gov NCT06403345; https://clinicaltrials.gov/study/NCT06403345

## Introduction

### Background

Reducing sedentary behavior and physical inactivity has been identified as an important modifiable approach to reduce risk for dementia [1,2]. Physical inactivity is 30% higher for older adults, people with disabilities, including people living with mild cognitive impairment (MCI) and dementia. Additionally, 31% of individuals who identify as Hispanic or Latino do not meet physical activity guidelines [3] and are 1.5 times more likely than non-Hispanic White individuals to develop dementia [4]. Hispanic or Latino individuals may face barriers to physical activity, such as a lack of sidewalks in their neighborhoods, unsafe streets and parks, and a lack of time and social support [3].

Although an active lifestyle is a key modifiable factor to lessen risk for developing dementia [5], Hispanic or Latino individuals are underrepresented in research to design and test physical activity interventions [6,7]. A promising approach is developing interventions that tap into the goals, preferences, and values of underrepresented populations is the use of physical activity in the context of nature.

A concern with existing physical activity interventions is that they are difficult to sustain [8,9], and often lack input from the end user in the development process. Most of these interventions have not been rooted in a human-centered approach that can iteratively account for the values and preferences of end users [10]. This is particularly the case for underrepresented populations such as Hispanic or Latino groups, who are estimated to rapidly increase in dementia incidence from 430,000 in 2012 to 2,383,000 in 2050 [4]. The purpose of this paper is to show the utility of the human-centered innovation biodesign (IB) framework [11] to develop and test interventions. Specifically, we apply the framework to develop and pilot the Nurturing Aging Through Uplifting Activities in a Restorative Environment (NATURE) program for Hispanic or Latino groups.

### NATURE Program

The NATURE program, formerly called the Green Activity Program, was co-designed with individuals who identified as Hispanic or Latino with MCI as a precursor to Alzheimer disease (AD) and early-stage dementia, called memory challenges herein, based on participants’ preferences [12]. NATURE promotes an active lifestyle in a social context through the skillful tailoring of local nature activities that a person enjoys and is delivered by occupational therapists. Furthermore, the intervention was designed with input from multiple partners, including local health care providers and nature organizations. Mapping the development of NATURE can provide a clear roadmap for incorporating human-centered design principles in the ongoing pilot study and provide a framework to depict additional refinements for future efficacy and effectiveness testing, and address challenges with sustainability faced by other physical activity interventions [8,9].

### The IB Framework

The IB framework uses a human-centered design approach. Human-centered design is an umbrella term for a flexible and replicable approach to innovation that prioritizes human values and experiences when creating and implementing complex systems, services, or products [10]. While there is disagreement about what “human” means, key design principles include placing the human experience at the center of the design, understanding the demands on users when planning tasks, and considering the surrounding environment. Human-centered design involves the end user in every step of the process and applies user-centered evaluation to iteratively refine the design [10,13]. These principles are applied across disciplines and sectors, from developing technology and mobile health to health care programming to promote behavior change. One critique of human-centered design is that it uses broad methods of engagement and can lack systematic processes to ensure rigor and quality. Particularly, end user engagement can occur through observations or more structured co-design with the end user’s input through participatory design sessions, town halls, focus groups, or individual interviews [14]. Using a framework can help mitigate this with a systematic process to map the application of human-centered design principles across the development and testing of an intervention.

Human-centered design principles within the IB framework enable researchers to identify and solve the unmet needs of underrepresented populations [11]. The framework has been applied to unmet clinical needs for asthma in minoritized populations [11] and can help map the development and testing of health care innovations, technologies, and interventions through collaborative partnerships. Collaborations between researchers, health care professionals, and those living with dementia in the design and implementation of an intervention may promote acceptability and applicability in addressing a population’s needs [14]. Here, we apply the framework to the development of an intervention to promote physical activity for Hispanic or Latino individuals with low resources.

The IB framework has three domains the (1) problem space, (2) invention, and (3) solution space [11]. Each domain includes activities that may not always occur in a linear process. The problem space involves a needs assessment, needs screening, evidence-based literature review, review of existing models of programs, and iterative feedback from partners, leading to an invention. The solution space comprises of implementation and validation of the invention and assessment of outcomes (planned or actual). The framework enables researchers to identify unmet needs through clinical observation and patient feedback and iteratively design, refine, and test user-centered solutions from development to dissemination.

Accordingly, we sought to apply the IB framework to map the development and piloting of the NATURE program to address the unmet needs of Hispanic or Latino individuals with memory challenges. This paper focuses on mapping the development of NATURE onto the problem space of the framework and uses the solution space to map a plan for an ongoing pilot study.

## Methods

### Study Design

In this use case, we applied the IB framework to map the development of the NATURE program and ongoing pilot study. Use-case findings are reported in adherence with the guidance for the reporting of intervention development checklist for reporting intervention development studies (Multimedia Appendix 1) [15].

This use case took place in the Bronx, New York; Indianapolis, Indiana; and Burlington, Vermont. NATURE, formerly the Green Activity Program, was initially designed for the Bronx [12]. The intervention was expanded with input from Chinese Americans and Black individuals. The focus of this use case is on the initial development in the Bronx, with Hispanic or Latino groups.

### Recruitment

We used convenience sampling to recruit participants with memory challenges from local aging organizations in the Bronx, Indianapolis, and Burlington. We also used direct referrals from 2 geriatric clinics in the Bronx, and Indianapolis. Inclusion criteria for participants from secondary data were previously reported [12]. Due to the current political climate in the United States, we expanded our population to include all races and ethnicities due to safety concerns for Hispanic or Latino individuals. Recruitment efforts were focused on areas with lower resources, with strategies of recruiting through in-person events, newsletters through local aging organizations, and clinical referrals.

Participants in the ongoing pilot study were (1) 45 years or older, (2) had subjective cognitive decline, and (3) had access and ability to respond to the telephone (mobile or landline). Subjective cognitive decline was determined by two questions: (1) Have you noticed difficulty with your memory? (2) Do you think that your memory is worse than 5 years ago? [16] Subjective cognitive decline was also characterized after enrollment with the Telephone Montreal Cognitive Assessment.

Participants were excluded if they had greater than 3 hospitalizations in the past year, or if they indicated any of the following on the 2023 Physical Activity Readiness Questionnaire +: (1) heart failure or difficulty controlling coronary artery disease, diagnosed abnormality of heart rhythm, or other cardiovascular condition; (2) cancer and are actively receiving therapies, (3) experienced a black out, fainted, or lost consciousness as a result of a head injury in the past 12 months, or (4) often experienced signs and symptoms of low blood glucose (hypoglycemia) following exercise or daily activities.

### Analysis: Steps to Apply the IB Framework to NATURE

We used a 3-step approach to apply the IB framework to NATURE. Step 1 involved identifying the problem to map onto the problem space. These sources included findings from other nature programs, reviews of the literature to identify existing interventions and surmise gaps, as well as focus groups to identify unmet needs. Focus groups occurred between March 2023 to August 2023. Focus groups included iterative co-design to design programmatic components, such as identifying local nature activities, program frequency, duration, and preferred outcomes, and identifying potential challenges and solutions for Hispanic or Latino individuals with memory challenges. Research design included identifying the preferred language for recruitment materials and referral pathways within a local health care system. In step 2, we describe the developed invention, the NATURE program. We used information from the pilot study protocol and intervention manual to describe NATURE. Step 3 encompassed mapping the ongoing pilot study. We used the protocol and planned data collection, including activity tracker technology, in the solution space to create a plan for an ongoing feasibility pilot study (ClinicalTrials.gov NCT06403345).

### Ethical Considerations

Secondary data from the initial study of NATURE (formerly the Green Activity Program) was approved by NYU (New York University) Langone (s22-01409) and Indiana University-Bloomington’s Institutional Review Boards (20660). Primary data collection for the feasibility study that is underway was approved by Indiana University-Bloomington’s Institutional Review Board (22206). Participants in both studies provided either written or verbal consent to participate. To protect confidentiality, study data were deidentified in both studies. Participants were compensated US $50 to participate in co-design sessions (secondary data) and US $225 to participate in the feasibility study (primary data study protocol). Applying human-centered design principles enabled participation from participants with varying abilities. We maintained confidentiality at every stage of intervention development and elicited feedback and participants at each stage to improve the intervention. No artificial intelligence technology was used in the writing or preparation of this paper.

## Results

**Figure 1:**
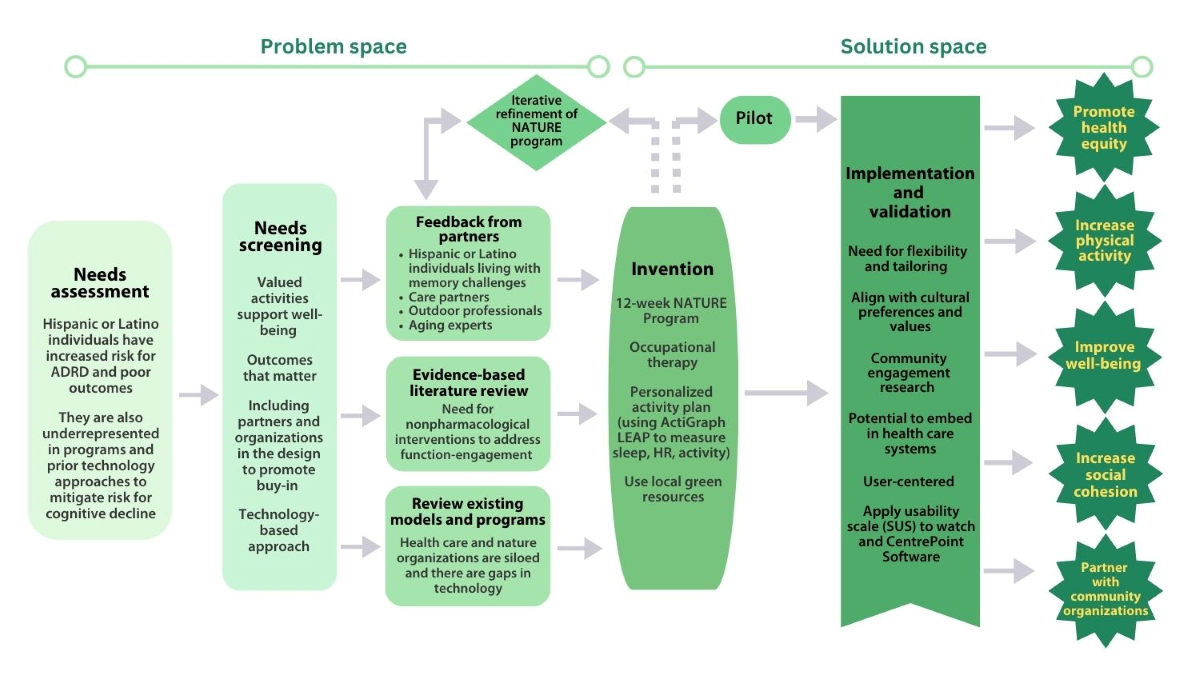
Applying the Innovation Biodesign Framework to map the development and piloting of the NATURE Intervention. The pilot study is underway in the Bronx, NY; Indianapolis, IN; and Burlington, VT. ADRD: Alzheimer disease-related dementias; HR: heart rate; NATURE: Nurturing Aging Through Uplifting Activities in a Restorative Environment; SUS: System Usability Scale.

We applied the IB framework in 3 steps (Figure 1).

### Step 1: Identifying the Problem (Problem Space)

#### Needs Assessment: Review of the Literature and Existing Programs

We identified a need for nature programs to meet the unmet need of physical inactivity and related risks of decreased well-being, dementia, and subsequent cognitive decline for Hispanic or Latino individuals living with memory challenges.

#### Physical Inactivity, Sedentary Behavior, and Dementia Risk

Physical inactivity is defined as failing to meet physical activity guidelines [17] and is attributed with 2% of the risk for developing dementia [1]. However, physical activity can also impact other modifiable risk factors, such as decreasing depression (3% risk reduction), hypertension (2%), and high low-density lipoprotein (7%) in midlife, and social isolation (5%) later in life [1], which collectively can contribute to a 19% reduction in the risk for developing dementia. In fact, the American Heart Association endorses physical activity as a critical component of a lifestyle approach as a first line of treatment for hypertension and high low-density lipoprotein [18]. Additionally, a recent meta-analysis with 191,130 participants found that if adults met physical activity guidelines, 11.5% of depression cases could be prevented [19].

Yet, physical activity alone is not enough to reduce risk for dementia and cognitive decline. A longitudinal study of 404 community-dwelling older adults who were free from dementia at baseline found that despite having high levels of physical activity, individuals with high sedentary behavior had neurodegeneration and worse cognition over 7 years [2]. Individuals with apolipoprotein E ε4, a well-established genetic risk factor for AD [20], were associated with high sedentary time and demonstrated faster hippocampal atrophy and decline in naming and information processing speed despite being physically active [2].

Sedentary behavior is an important risk factor for dementia [2]. Sedentary behavior is defined as exerting 1.5 metabolic equivalents in sitting, reclining, or lying down [17]. The World Health Organization recommends replacing 30 minutes of sedentary behavior a day with any kind of physical activity [21]. Replacing 30 minutes/day of leisure-time sedentary behavior with an equal amount of time with different types of physical activity was associated with a 7%-18% decreased risk of dementia incidence and a 12%-21% risk reduction in mortality from dementia for adults with a diagnosis [22].

#### Nature and Physical Activity

One promising approach to reduce sedentary time and promote physical activity is using nature activities that a person enjoys and are meaningful to them. Nature activity is physical activity in contact with nature. Nature activity can lower anxiety more than physical activity without contact with nature (indoors or outdoors) [23,24]. Simply visiting a park or natural space for 30 minutes at least once a week can reduce hypertension and depression by 7%-9% [25], which are risk factors for dementia [1]. Moreover, visiting parks or green spaces is also associated with higher physical activity and social cohesion [25].

#### Review of Existing Nature Programs

Nature or “green” prescriptions are integrated into health care systems in New Zealand, Australia, and the United Kingdom for middle-aged and older adults to reduce risk for cardiovascular disease, diabetes, and improve mental health [15,26,27]. Green prescriptions describe the process of prescribing nature experiences or activities to improve a person’s health and well-being. Green prescribing provides an enjoyable means to engage in physical activity at a low cost and is rapidly growing [15,26-31]. These interventions are particularly beneficial for people who lack access to nature [29] or experience lower resources [32]. However, there are many different types of nature prescriptions that are implemented in varied settings and with different populations. Components of these prescriptions show promise for people at risk for or living with dementia (Table 1).

**Table 1:** Examples of nature prescription programs and models.

Type	Setting or delivery	Population	Benefits	Challenges
Green exercise: New Zealand [15,28,33,34]; Australia [27]	Primary care, nurse, and exercise specialist	Inactive middle-aged, older adults [33]	Physical activity, cognition, QoL^a^ [34], safe, effective [33], and sustained change [15]	Time, referrals [35], and adherence are impacted by institutional policies and local resources [36]
Green social prescribing [26,31,37,38]: United Kingdom	Primary care and community worker	Mild to moderate mental health problems, community-dwelling adults	Health, well-being, equity, cost-effective, and sustainable [38]	Infrastructure [31], low referrals, appropriateness of referral, and terminology [26]
Gardening [39-42]	Community, long-term care, recreation staff (eg, horticulture therapist), and OT^b^	AD/ADRD^c^, MCI^d^, care partners	Well-being, engagement, social, cognitive function [39], decreased BPSD^e^ [39-42]	Falls, wheel-chair accessibility, and tailoring activities
Animal-assisted interventions [43-46] and farms	Community, long-term care, recreation staff, OT’s, physical therapists, and nurses	AD/ADRD, MCI, care partners	Engagement, well-being, cognitive function, social interaction [43], and decreased BPSD [44-46]	Tailoring activities and environmental modifications to avoid safety risks (eg, falls)

^a^QoL: quality of life.

^b^OT: occupational therapist.

^c^AD/ADRD: Alzheimer disease and Alzheimer disease disease-related dementias.

^d^MCI: mild cognitive impairment.

^e^BPSD: biopsychosocial symptoms of dementia.

#### Outcomes of Nature Prescription Programs

As depicted in Table 1, nature prescriptions for other populations come with potential benefits and challenges. Benefits include increased physical activity, health, cognition, and well-being with sustained behavior change for middle-aged and older adults. While nature prescriptions have not been studied for populations with dementia, nature activities involving gardens and animals add potential benefits of reduced behavioral symptoms (eg, anxiety or depression) and improve well-being [44-46]. Moreover, nature prescriptions contain beneficial components of social engagement, nature, and meaningful activities, which can support function and stave off decline in persons living with dementia [47,48]. Plausible explanations for positive outcomes include (1) regulation of immunological and physiological (stress) responses, (2) enhancement of psychological states (mood and attention), and (3) facilitation of health-promoting behaviors (exercise and social contact) [36], with a central biological pathway of immune functioning by Kuo [49] encompassing all. Despite the potential benefits, a formal nature activity prescription program for this population has yet to be developed and tested.

#### Identification of Gaps: Challenges, and Critiques of Nature Programming

Despite the benefits, there are challenges to implementing nature programs. Challenges include appropriateness of the activities to meet the person’s needs, institutional policies, and a lack of available natural (green) resources, as contributing to low referral rates in primary care [36]. Difficulties with participant engagement also present a challenge to implementing nature prescriptions, as many of these programs grew from a bottom-up approach in the United Kingdom, resulting in challenges with buy-in [15,50].

Critiques of current nature prescriptions include challenges with referrals in primary care settings, varying terminology [36], and overlooking groups who have historically been excluded from research. Limitations of nature prescriptions include a lack of testing in persons living with dementia and a lack of culturally congruent programs for individuals who have been minoritized in the United States. A recommended strategy to engage underrepresented groups in physical activity interventions is to promote social cohesion and use local resources to increase participation and sustain behavior change [51].

#### Identification of the Problem

Despite the potential benefits of nature activities to improve well-being and reduce risk for dementia and cognitive decline after a dementia diagnosis, a comprehensive nature activity program for this population has not been developed. Prior models of nature programs have often overlooked communities that are minoritized in the United States, who are at higher risk for developing dementia, such as Hispanic or Latino populations [4]. Additionally, most nature prescribing models are commonly implemented with community workers, who are often unequipped to tailor activities to a person’s fluctuating levels of function and decline associated with dementia [52]. Occupational therapists are uniquely positioned to address this need as tailoring and matching valued activities to a person’s function, preferences, and needs are central to their scope of practice [53]. There is a need to design an occupational therapist-led comprehensive nature activity program to safely tailor and implement nature activities for people with MCI as a precursor to AD and those living with dementia.

A nature program has not been developed for Hispanic or Latino individuals with memory challenges in the United States, and culturally adapted programs for this population in general are scarce [54]. There is a need to develop a comprehensive nature program that prioritizes the values and human experiences of Hispanic or Latino people living with memory challenges. In line with human-centered design principles, key Hispanic or Latino values that impact the use of supportive programming include familismo (responsibility to family), personalismo (relationships with others), respeto (respect), and dignidad (dignity and self-respect) [55].

Additionally, there is a need to use a partner-driven approach to develop a nature-prescribing program. Particularly, prior nature prescribing programs have lacked input from Hispanic or Latino individuals with memory challenges, care partners, local health care providers, and outdoor organizations. Using a partner-driven approach to develop and implement a nature intervention could mitigate challenges with buy-in and cohesion [36]. To address these gaps, the NATURE program was designed with input from multiple partners, including Hispanic and Latino individuals with memory challenges, care partners, outdoor professionals, and health care providers.

#### Need Screening: Feedback From Partners and Iterative Refinement

Feedback from partners was elicited during 5 focus groups and 2 interviews to co-design the NATURE intervention. End user engagement through iterative co-design was as described in Lassell et al [12] to inform key elements of the program and research design of the ongoing pilot study. Co-design occurred with Hispanic or Latino individuals living with memory challenges, care partners, outdoor professionals, and interdisciplinary health care providers using a community-based participatory action research [56] approach with human-centered design principles. A community-based participatory action research approach involves an iterative cycle of acting, assessing, reflecting, and refining. People living with memory challenges and their care partners were empowered to contribute to each phase of the research process. Community-based participatory action research principles of empowerment, capacity building, and resource use were applied throughout the development and implementation process. Actions supporting these principles occurred with identifying outcomes relevant to community partners and end users (all groups) with program co-design, implementing the protocol, assessing its acceptability and feasibility, and refining based on their input.

Co-design enabled key elements of the program to align with participant preferences and needs. Co-designed elements of the research design were recruitment strategies, including preferences for terminology and language from the end users (eg, people living with memory challenges vs dementia and Hispanic or Latino vs Latinx). The NATURE program was designed to align with the cultural preferences and values of the Hispanic or Latino community. Particularly, the values of the design of the program also incorporated Hispanic or Latino values of respeto and dignidad were built into the occupational therapy assessment by asking what matters most to the person. Additionally, the values of personalismo and familismo were incorporated with options to include family or friends in the Nature Activity Plan and intervention sessions [12].

End users of Hispanic or Latino people living with memory challenges identified a range of nature activities they enjoyed (eg, walking, dancing, or feeding squirrels) and preferences for program frequency, duration, and delivery-based on prior evidence-based programs [15,25]. They underscored the need to offer a range of options for flexible participation that could be tailored according to individual needs and preferences (eg, 4-8 sessions between 30-90 minutes long). Outdoor professionals provided input on local nature activities and how they would prefer to be supported by the occupational therapist to participate in the program. Health care providers advised on clinical referral pathways, including primary care and community-based organizations. See Lassell et al [12] for iterations of the program before and after the co-design process.

### Solution Space

#### Step 2: Developing the Invention NATURE

The NATURE intervention was informed by the Theory of Cognitive Reserve [57], the Attention Restoration Theory [58], and the environmental press model by Lawton [59], with the assumption that tailoring nature activities that are meaningful to a person (matching the activity demands to their context, preferences, and needs) can support a person’s participation in nature activities and well-being. The co-design process incorporated options for session duration and frequencies based on previously successful programs such as the Tailored Activity Program for people living with dementia [60-62] and 2 green prescribing models [15,63]. Co-design resulted in the creation of a 12-week NATURE intervention that incorporated the preferences and needs of Hispanic or Latino and care partners, local outdoor professionals, and interdisciplinary health care providers [12].

The goal of the NATURE intervention is to promote an active lifestyle and well-being by mitigating risk for dementia and cognitive decline. The intervention advances the US Department of Health and Human Services “National Plan to Address Alzheimer’s Disease” Goal 3 [64] as a support for people living with AD and their families and is driven by outcomes that mattered to Hispanic or Latino individuals living with memory challenges [12]. The program involved an occupational therapy assessment, goal-setting, and a tailored green activity plan that could be built into a person’s daily routine to address physical inactivity and sedentary time throughout the day. The program emphasizes using local community resources to increase access for homebound participants and to promote sustainability (Table 2).

**Table 2:** NATURE^a^ program for people living with memory challenges.

Time	Description
Week 1: visit 1	OT^b^ evaluation (90 minutes): clinical interview, daily habits and routines, function, fall risk, nature activity checklist, and goal attainment scaling Education: depression and local resources (eg, food, housing, and transportation). Benefits of nature activities. Participants received a nature activity program binder with educational materials, study contact information, weekly planning behavioral activation worksheets, and a whiteboard weekly calendar
Week 2: visit 2	Personalized nature activity plan (30-90 minutes) Finish assessments from the evaluation. Observe or test 1 green activity together. Update plan as needed Education: SMART^c^ goal setting, strategy training, and behavioral activation
Week 3	Option for additional visit; follow the nature activity plan
Week 4: visit 3	Test group green activity (30-90 minutes) Adjust green activity plan as needed Education: fall prevention, strategy training, and green activity plan
Week 5	Option for additional visit; follow the nature activity plan
Week 6: visit 4	Test third green activity (30-90 minutes) Adjust plan as needed Education: fall prevention and strategy training
Week 7	Option for additional visit; follow the nature activity plan
Week 8	Support from OT: phone or Zoom (Zoom Communications, Inc.) check-in (20-30 minutes)
Week 9	Option for additional visit; follow the nature activity plan
Week 10	Support from OT: phone or Zoom (Zoom Communications, Inc.) check-in (20-30 minutes)
Week 11-12	Follow the nature activity plan

^a^NATURE: Nurturing Aging Through Uplifting Activities in a Restorative Environment.

^b^OT: occupational therapy.

^c^SMART: Specific, Measurable, Achievable, Relevant, and Time-Bound.

#### Notes

Function was assessed with the Functional Activities Questionnaire, fall risk. The Fall Risk Assessment Tool and the World Health Organization fall risk clinical algorithm were conducted by a trained research assistant before the program. Individuals who were at high fall risk were required to have a care partner present for program sessions and green activities.

NATURE was implemented by 2 licensed occupational therapists with a minimum of 4 years of experience in geriatrics and dementia care. The occupational therapists were trained using a training manual of NATURE with templates for session notes and evaluation forms. The occupational therapists delivered NATURE either virtually using a secure Zoom platform or in-person, based on participant need and preference, in alignment with co-design [12]. NATURE involves 3 phases: assessment, coaching, and self- or care partner–monitoring and sustained behavior change.

The assessment phase informed how the nature activities were tailored in a participant’s NATURE plan (Table 3). The assessment phase involved 2 sessions, ranging from 60-90 minutes. Based on the assessment which included clinical interviews, functional assessments, and validated questionnaires, tailoring occurred on 3 levels: the person and family, the nature activity, and their daily contexts (environmental modifications based on accessibility, safety, weather, equipment needed, level of social support, socioeconomic resources, and the nature resources available to the person in their home, neighborhood, and community). As part of the assessment, participants engaged in collaborative goal-setting using Goal-Attainment Scaling to develop 3 goals for 3 separate individual nature activities that the person wanted to do. Local nature activities were selected from a list compiled through co-design [12]. The local nature list included cost and whether there were virtual options. The participant worked with the occupational therapist to select 3 nature activities they would prefer to do. Goals to engage in the 3 selected nature activities were based on the person’s level of function and incorporated the World Health Organization’s guidelines for physical activity. Goals could be made to engage in a nature activity or to safely access a nature activity (eg, learning a new bus route to a park) and had options for good days and hard days, and virtual options for inclement weather.

**Table 3:** Areas of assessment and tailoring in the NATURE^a^ program.

Areas			Description
**Assessment**			
	Personalized delivery	PROMIS^b^ health, mental health, and social participation UCLA^c^ Loneliness Scale Geriatric depression screening Neuropsychiatric Symptom Questionnaire Behavioral Activation Scale Nature-Connectedness Scale (also relevant to context) Clinical interview A: background Clinical interview B: routines and preferred nature activities (also context) Comportment Scale	
	Activity modification	Activity levels (Quick Physical Activity Questionnaire) Physical Activity Readiness Questionnaire + (includes relevant comorbid conditions) Telephone Montreal Cognitive Assessment Functional Activities Questionnaire Function executive function screen	
	Context	Health-related social needs Fall risk assessment (also relevant to personalized delivery) Environmental assessment: modified Wemstead home assessment Clinical interview B: routines and preferred nature activities Nature-Connectedness Scale (also relevant to context)	
**Tailoring**			
	Personalized delivery	Incorporated the person and family’s strengths, interests, and values, and honored dislikes. Options for adaptation based on the person’s level of function Personalized with the level of supervision and cueing (verbal, visual, and physical) needed for safety How the nature activity was delivered: individually or with family, or with friends	
	Activity modification	Options and strategies to make an activity harder or easier for good and challenging days Activity adaptations included: the number of steps in directions, frequency, and duration of the activity, and the number of rest breaks; included options for backward chaining and forward chaining Backward chaining Forward chaining	
	Context	Environmental modifications (eg, raised garden beds or tabletop gardening activities) Level of safety (eg, neighborhood crime rates, environmental hazards, and accessibility) Weather Accessibility of sidewalks, parks, and natural spaces Toxic land use and pollution Social support Socioeconomic resources Available natural resources in a person’s home, neighborhood, and community	

^a^NATURE: Nurturing Aging Through Uplifting Activities in a Restorative Environment.

^b^PROMIS: Patient-Reported Outcomes Measurement Information System.

^c^UCLA: University of California Los Angelos.

The coaching phase involved either 1:1 virtual, phone, or in-person visits with a trained and licensed occupational therapist lasting between 30 and 90 minutes. The occupational therapist developed a personalized NATURE plan for each participant. Care partners were invited to join but were not required unless a participant was at high fall risk or if they required a legally authorized representative to attend. Each session involved the following components: greeting or introduction, reviewing progress toward each goal, testing at least 1 nature activity related to their goal, making adjustments as needed, reviewing the weekly behavioral activation worksheets, planning their nature activities for the next week, educational materials (nature activities, depression, reduction of fall risk during plan, and participant-specific education), reviewing session points and scheduling the next session. Behavioral activation worksheets involved weekly goal-setting using SMART (Specific, Measurable, Achievable, Relevant, and Time-Bound) goals, planning their activity, telling their team (occupational therapist, family, or friend), reflecting on how the nature activity went, and problem-solving with the occupational therapist afterward. Care partners completed the behavioral activation form for individuals who had greater cognitive impairment. Participants also mapped out their nature activities for each week using a dry-erase calendar. They planned their nature activities embedded into their daily schedules while considering the weather and air quality. There was an option to include an additional 3 in-person or virtual visits as needed, based on participant need.

The self-monitoring phase involved 2 phone check-ins that were at least 15 minutes with the occupational therapist. Check-in sessions involved a greeting, discussing progress toward goals, and problem-solving any challenges that arose with their NATURE plan, with additional adjustments as needed. Additional education was also provided in relation to each participant’s NATURE plan.

#### Step 3: Mapping the Pilot Study and its Implementation and Validation

NATURE sessions are being implemented virtually or in-person with 1:1 sessions by a licensed occupational therapist. Two occupational therapists are implementing NATURE and received 4 hours of training guided by the NATURE training manual.

The pilot study is being implemented through continued community engagement with local aging organizations that provide services in the Bronx; Indianapolis; and Burlington. Participant feedback regarding program feasibility (retention, feasibility of outcome measures, or fidelity) and acceptability (questionnaires and interviews) is being gathered. Planned outcomes of the program include promoting health equity and an active lifestyle, mitigating risk factors for cognitive decline related to physical inactivity and social isolation. Additionally, health outcomes related to self-reported physical activity using the Quick Physical Activity Rating [65] scale and well-being, such as the PROMIS measures of Social Participation, Mental Health, and Physical Health [66,67], are also being collected among other health outcomes (Table 4).

**Table 4:** Health outcomes.

Primary goal	Goal attainment scaling (primary goal met, yes or no)
Function	Telephone Montreal Cognitive Assessment, Quick Physical Activity Rating Scale, Functional Activities Questionnaire, Physical Activity (ActiGraph)
Health	Heart rate, sleep (ActiGraph), PROMIS^a^ (mental health, physical health, and social participation), Geriatric Depression Scale Short form, UCLA^b^ Loneliness Scale, Neuropsychiatric Symptoms Questionnaire
Nature	Nature Connectedness Scale
Usability	Holden Systems’ Usability Scale [68]
Mechanism of change	Behavioral Activation Short-Form

^a^PROMIS: Patient-Reported Outcomes Measurement Information System.

^b^UCLA: University of California Los Angeles.

Using human-centered design principles, we are incorporating multiple methods to measure preferred outcomes of well-being, sleep, and social connection. We are using the ActiGraph LEAP to measure physiological data related to well-being (heart rate and activity), sleep, and validated questionnaires. We are also piloting the CentrePoint Connect mobile app (ActiGraph, LLC) sync ActiGraph data in real-time and assess the usability of these technologies using Holden Systems’ Usability Scale [68]. Additionally, qualitative data will be gathered to learn about each participant’s experience during the program to iteratively refine the intervention and measurement of outcomes before efficacy testing.

The NATURE intervention is designed to be sustainable through its use of local resources that are accessible by participants (eg, ongoing nature activities in their community, recommending nature activities they can still access after the intervention) with tailored strategies to adapt and integrate nature activities into the person’s daily routine. Using local natural resources may also contribute to social cohesion for participants and their communities. This can be achieved through continued partnerships with local outdoor organizations and our local health care partners.

If determined effective in the future, the NATURE intervention has the potential to be embedded into current health care systems, with potential reimbursement under Medicare with skilled home health care (part A) and home-based outpatient services covered under part B [69]. However, before being implemented in other contexts with other groups, context-specific adaptations will need to be made by identifying local nature activities and referral pathways for local community-based organizations and hospital systems. Additional refinements may also be needed for language preferences to describe AD and related dementias to successfully recruit participants from other cultural groups, and have members of the group assist in implementation. Additional adaptations may also be needed to align with cultural values.

## Discussion

### Overview

The main finding of this use case is that the IB framework provides a systematic approach to apply human-centered design principles to design a program to address well-being and physical activity in Hispanic or Latino individuals with a range of cognitive abilities. The application of the IB framework [11] use case of the NATURE [12] intervention highlights the utility in visualizing the application of human-centered design principles throughout NATURE’s progression from development to piloting. The framework will continue to guide human-centered refinements through efficacy and effectiveness testing and dissemination as the program develops and can serve as a useful tool for other researchers seeking to develop human-centered programs for underrepresented populations.

### Principal Results

The IB framework [11] enabled us to map human-centered design principles in end user engagement through the application of co-design in creating the NATURE [12] program to be responsive to the preferences and needs of Hispanic or Latino people living with memory challenges. It also provided a map to guide piloting an activity tracker with a mobile app as a means to measure sleep and outcomes related to well-being (heart rate and activity) and assess the usability of these technologies. Use of the IB framework and other human design frameworks can advance health equity by prioritizing diverse participants’ needs and experiences [10,11].

The current study underscores the benefits of using the IB framework to develop and test interventions and programs with input from multiple partners, as well as the end user. This aligns with the human-centered design principle of involving the user and multiple partners in every step of the design process and understanding the context in which the intervention or program will occur [10,13]. The IB framework provided guiding principles for moving the challenge of addressing physical inactivity in Hispanic or Latino older adults with memory challenges from a problem space to a solution space informed by the needs and preferences of partners and participants [11]. The inclusion of multi-partner perspectives is an inclusive innovation process that values input from all involved, including older adults living with memory challenges.

Rapid-cycle iterations within the IB framework enable researchers to map human-centered involvement and adapt a program or innovation based on participants’ dynamic needs [11]. For example, the rapid-cycle iterations that occurred during the co-design of NATURE enabled researchers to update the program in response to participant feedback and insights. Rapid-cycle iterations can help NATURE and other health innovations successfully evolve based on the end users’ dynamic preferences and needs, enhancing the program or products’ accessibility, impact, and reach. This aligns with human-centered design principles of rapid-cycle iterations and refinements based on the end user’s evaluation [10,13]. Next steps for NATURE are to finish piloting the intervention and receive input from the end users (participants and partners) on refinements. Once proof of concept and feasibility have been established, the next steps include effectiveness testing and maximizing the outreach of the program.

### Comparison With Prior Work

While there are other helpful human-centered design frameworks, they are not specifically designed to incorporate the voices of underrepresented populations. For example, the Approach to Human-Centered, Evidence-Driven Adaptive Design for health care interventions focuses on an empathy-driven approach to rapidly design health care innovations [70] and does not focus on the inclusion of minoritized groups in the design process. Additionally, the discover, design or build, and test framework draws from other evidence-based human-centered design frameworks to modify and evaluate the usability of evidence-based psychosocial interventions and implementation strategies [71]. A benefit of using the IB framework was that it allowed us to incorporate the voices of Hispanic or Latino individuals to design NATURE and assess the usability of an activity tracker and mobile app technology to measure and monitor outcomes related to sleep, heart rate, and activity.

Benefits of using the IB framework from prior research include improved health and digital health literacy for patients with asthma [11]. Through its collaborative approach, this framework has improved clinical outcomes for individuals with asthma but also enabled health care providers to offer more personalized care plans. Patients were better equipped to understand and manage their health conditions through education provided by an app. The framework fosters stronger patient-provider relationships, bolstering trust and communication. For individuals with memory challenges, active involvement in this framework could enhance their grasp of health issues, treatment choices, and self-care strategies. This empowerment may lead to better adherence to treatment and overall well-being, emphasizing a patient’s proactive role in health management and aligning with human-centered design principles of prioritizing the human experience [13]. Empowering patients as the end user can be critical when designing health interventions and innovations that seek to promote health equity [10].

The framework can also be helpful for health care providers as it fosters the development of cutting-edge medical technologies and solutions tailored to address unmet clinical needs [11]. By integrating engineering, business, and medical disciplines, the framework equips providers with the tools to innovate and improve patient care outcomes effectively. This aligns with the human-centered design principle of using a multidisciplinary team [13]. The framework is also highly relevant to multidisciplinary medical and clinical informatics professionals because it focuses on collaborative and inclusive health care innovation with a systematic process to map human-centered design. Public health professionals may also find this framework helpful to develop policies and public health initiatives by using human-centered design principles, such as the end users’ evaluation to iteratively refine a policy for better uptake [10]. This process can guide the development of programs, interventions, and innovations that are specifically designed to meet the needs of diverse populations, which can increase their effectiveness and acceptance.

Application of the IB framework provided a structure for applying human-centered design principles to design and pilot the NATURE program for Hispanic or Latino individuals with memory challenges. The framework can be applied to the development and implementation of other interventions to incorporate the human experiences and values of the end user and may support the applicability and sustainability of interventions for underrepresented populations.

### Limitations

A limitation of applying the IB framework in the NATURE use case is that the framework spans development through the dissemination phases of an intervention or program. Our use case is focused on a program that is in the early piloting phase of development and does not provide the full range of the framework through efficacy testing and dissemination. Yet, a strength of our use case is that it shows the utility of using the framework in the early stages of intervention development, which can guide feasibility and piloting through efficacy testing and dissemination. A limitation of using secondary data was that it was initially collected for another purpose (designing NATURE) and was retroactively fitted to the IB framework. Yet, a strength of our secondary data was that it matched the user-centered design process of the IB framework.

## Data Availability

Data sharing does not apply to this paper as no datasets were generated or analyzed during this study.

## Appendix

Multimedia Appendix 1 GUIDED checklist.

## Abbreviations

AD: Alzheimer disease
IB: innovation biodesign
MCI: mild cognitive impairment
NATURE: Nurturing Aging Through Uplifting Activities in a Restorative Environment
SMART: Specific, Measurable, Achievable, Relevant, and Time-Bound
