# Supplementary material for: Applying the Human-Centered Innovation Biodesign Framework to the Development and Piloting of a Program to Mitigate Risk for Cognitive Decline Among Historically Underrepresented Individuals: Case Study
Source: JMIR Form Res. 2025 Oct 15;9:e64930. doi: 10.2196/64930 (PMC12572745; doi:10.2196/64930)
Supplement: Multimedia Appendix 1 [file formative_v9i1e64930_app1.pdf]

GUIDED – a guideline for reporting for intervention development studies.

Multimedia Appendix 1: completed checklist

Item description	Explanation	Page in manuscript where item is located	Other*
1. Report the context for which the intervention was developed.	Understanding the context in which an intervention was developed informs readers about the suitability and transferability of the intervention to the context in which they are considering evaluating, adapting or using the intervention. Context here can include place, organisational and wider socio-political factors that may influence the development and/or delivery of the intervention (15).	The context of the intervention development is detailed in Step 1 and Step 2. The needs assessment and the developed intervention (pp. 4-13, 1st para).	
2. Report the purpose of the intervention development process.	Clearly describing the purpose of the intervention specifies what it sets out to achieve. The purpose may be informed by research priorities, for example those identified in systematic reviews, evidence gaps set out in practice guidance such as The National Institute for Health and Care Excellence or specific prioritisation exercises such as those undertaken with patients and practitioners through the James Lind Alliance.	"The goal of the NATURE intervention is to promote an active lifestyle and well-being by mitigating risk for dementia and cognitive decline. The intervention advances the US Department of Health and Human Services “National Plan to Address Alzheimer’s Disease” Goal 1868 as a nonpharmacological trial to treat dementia and is driven by outcomes that mattered to Hispanic/Latino individuals living with memory challenges.16 " p. 12, para 2.	
3. Report the target population for the intervention development process.	The target population is the population that will potentially benefit from the intervention – this may include patients, clinicians, and/or members of the public. If the target population is clearly described then readers will be able to understand the relevance of the intervention to their own research or practice. Health inequalities, gender and ethnicity are features of the target population that may be relevant to intervention development processes.	"This use-case took place in the Bronx, New York, Indianapolis, Indiana, and Burlington, VT. ... The intervention was expanded with input from Chinese Americans and Black individuals. The focus of this use-case is on the initial development in the Bronx, NY with Hispanic/Latino groups." p 5, para 2.	
4. Report how any published intervention development approach contributed to the development process	Many formal intervention development approaches exist and are used to guide the intervention development process (e.g. 6Squid (16) or The Person Based Approach to Intervention Development (17)). Where a formal intervention development approach is used, it is helpful to describe the process that was followed, including any deviations. More general approaches to intervention development also exist and have been categorised as follows (3):- Target Population-centred intervention development; evidence and theory-based intervention development; partnership intervention development; implementation-based intervention development; efficacy-based intervention development; step or phased-based intervention development; and intervention-specific intervention development (3). These approaches do not always have specific guidance that describe their use. Nevertheless, it is helpful to give a rich description of how any published approach was operationalised	**"Feedback from partners was elicited during 5 focus groups and 2 interviews to co-design the NATURE intervention. End-user engagement through iterative co-design as described in Lassell et. al12 to inform key elements of the program and research design of the ongoing pilot study. Co-design occurred with Hispanic/Latino individuals living with memory challenges, care partners, outdoor professionals, and interdisciplinary healthcare providers utilizing a Community Based Participatory Action Research (CBPAR)60 approach with human-centered design principles. A CBPAR approach involves an iterative cycle of acting, assessing, reflecting, and refining. People living with memory challenges and their care partners were empowered to contribute in each phase of the research process. Community-based participatory action research principles of empowerment, capacity building, and resource utilization were applied throughout the development and implementation process. Actions supporting these principles occurred with identifying outcomes relevant to community partners and end-users (all groups) with program co-design, implementing the protocol, assessing its acceptability and feasibility, and refining based on their input." (page 11, para 1)**	
5. Report how evidence from different sources informed the intervention development process.	Intervention development is often based on published evidence and/or primary data that has been collected to inform the intervention development process. It is useful to describe and reference all forms of evidence and data that have informed the development of the intervention because evidence bases can change rapidly, and to explain the manner in which the evidence and/or data was used. Understanding what evidence was and was not available at the time of intervention development can help readers to assess transferability to their current situation.	See Methods: "Step 1 involved identifying the problem to map onto the problem space. These sources included findings from other nature programs, reviews of the literature to identify existing interventions and surmise gaps as well as focus groups to identify unmet needs....nI Step 2, we describe the developed invention, the NATURE program. We used information from the pilot study protocol and intervention manual to describe NATURE." (P 5, para 1)<br>Results: Step 1. pp 7-11.	
6. Report how/if published theory informed the intervention development process.	Reporting whether and how theory informed the intervention development process aids the reader's understanding of the theoretical rationale that underpins the intervention. Though not mentioned in the e-Delphi or consensus meeting, it became increasingly apparent through the development of our guidance that this theory item could relate to either existing published theory or programme theory	"The NATURE program was informed by the Theory of Cognitive Reserve,41 Attention Restoration Theory,42 and Powell’s Lawton’s environmental press model,43 with the assumption that tailoring nature activities that are meaningful to a person (matching the activity demands to their context, preferences, and needs) can support a person’s participation in nature activities and well-being." p. 11-12	
7. Report any use of components from an existing intervention in the current intervention development process.	Some interventions are developed with components that have been adopted from existing interventions. Clearly identifying components that have been adopted or adapted and acknowledging their original source helps the reader to understand and distinguish between the novel and adopted components of the new intervention.	See response to answer 5.	
8. Report any guiding principles, people or factors that were prioritised when making decisions during the intervention development process.	Reporting any guiding principles that governed the development of the application helps the reader to understand the authors' reasoning behind the decisions that were made. These could include the examples of particular populations who views are being considered when designing the intervention, the modality that is viewed as being most appropriate, design features considered important for the target population, or the potential for the intervention to be scaled up.	"Co-design enabled key elements of the program to align with participant preferences and needs. Co-designed elements of the research design were recruitment strategies, including preferences for terminology and language from the end-users, (e.g., people living with memory challenges vs. dementia and Hispanic/ Latino vs. Latinx). The NATURE program was designed to align with cultural preferences and values of the Hispanic/Latino community. Particularly the values of The design of the program also incorporated Hispanic/Latino values of respeto and dignidad were built into the occupational therapy assessment by asking what matters most to the person. Additionally, the values of personalismo and familismo were incorporated with options to include family or friends in the Nature Activity Plan and intervention sessions.12 " (p 11, para 2)	

Item description	Explanation	Page in manuscript where item is located	Other*
9. Report how stakeholders contributed to the intervention development process.	Potential stakeholders can include patient and community representatives, local and national policy makers, health care providers and those paying for or commissioning health care. Each of these groups may influence the intervention development process in different ways. Specifying how differing groups of stakeholders contributed to the intervention development process helps the reader to understand how stakeholders were involved and the degree of influence they had on the overall process. Further detail on how to integrate stakeholder contributions within intervention reporting are available (19).	"End-users of Hispanic/Latino people living with memory challenges identified a range of nature activities they enjoyed (e.g., walking, dance, feeding squirrels) and preferences for program frequency, duration, and delivery based on prior evidence-based programs.27,29 They underscored the need to offer a range of options for flexible participation that could be tailored according to individual needs and preferences (e.g., 4-8 sessions between 30-90 minutes long). Outdoor professionals provided input on local nature activities and how they would like to be supported by the occupational therapist to participate in the program. Healthcare providers advised on clinical referral pathways including primary care and community-based organizations. See Lassell et al16 for iterations of the program before and after the co-design process." (p. 11, para 3).	
10. Report how the intervention changed in content and format from the start of the intervention development process.	Intervention development is frequently an iterative process. The conclusion of the initial phase of intervention development does not necessarily mean that all uncertainties have been addressed. It is helpful to list remaining uncertainties such as the intervention intensity, mode of delivery, materials, procedures, or type of location that the intervention is most suitable for. This can guide other researchers to potential future areas of research and practitioners about uncertainties relevant to their healthcare context.	"End-user engagement through iterative co-design as described in Lassell et. al12 to inform key elements of the program and research design of the ongoing pilot study. " (p 11, para 1) & "However, prior to being implemented in other contexts with other groups, context-specific adaptations will need to be made by identifying local nature activities and referral pathways for local community-based organizations and hospital systems. Additional refinements may also be needed for language preferences to describe Alzheimer's disease and related dementias to successfully recruit participants from other cultural groups and having members of the group assist in implementation. Additional adaptations may also be needed to align with cultural values." p. 14, para 3)	
11. Report any changes to interventions required or likely to be required for subgroups.	Specifying any changes that the intervention development team perceive are required for the intervention to be delivered or tailored to specific sub groups enables readers to understand the applicability of the intervention to their target population or context. These changes could include changes to personnel delivering the intervention, to the content of the intervention, or to the mode of delivery of the intervention.	See response 10.	
12. Report important uncertainties at the end of the intervention development process.	Intervention development is frequently an iterative process. The conclusion of the initial phase of intervention development does not necessarily mean that all uncertainties have been addressed. It is helpful to list remaining uncertainties such as the intervention intensity, mode of delivery, materials, procedures, or type of location that the intervention is most suitable for. This can guide other researchers to potential future areas of research and practitioners about uncertainties relevant to their healthcare context.	See response 10.	
13. Follow TIDieR guidance when describing the developed intervention.	Interventions have been poorly reported for a number of years. In response to this, internationally recognized guidance has been published to support the high quality reporting of health care? interventions[5]and public health interventions[14]. This guidance should therefore be followed when describing a developed intervention.	TIDer checklist items 1-2 see respone 6. See Table 2 for information from the TiDer checklist items 3-9. The pilot study is currently underway and items 10-12 will be reported with the findings.	
14. Report the intervention development process in an open access format.	Unless reports of intervention development are available people considering using an intervention cannot understand the process that was undertaken and make a judgement about its appropriateness to their context. It also limits cumulative learning about intervention development methodology and observed consequences at later evaluation, translation and implementation stages. Reporting intervention development in an open access (Gold or Green) publishing format increases the accessibility and visibility of intervention development research and makes it more likely to be read and used. Potential platforms for open access publication of intervention development include open access journal publications, freely accessible funder reports or a study web-page that details the intervention development process.	The co-design paper is published as open access and this paper will be as well.	

*e.g. if item is reported elsewhere, then the location of this information can be stated here.

2
